# Elevated galectin-3 levels detected in women with hyperglycemia during early and mid-pregnancy antagonizes high glucose − induced trophoblast cells apoptosis via galectin-3/foxc1 pathway

**DOI:** 10.1186/s10020-023-00707-5

**Published:** 2023-08-25

**Authors:** Yu Deng, Hongyan Jin, Jie Ning, Dong Cui, Muqiu Zhang, Huixia Yang

**Affiliations:** 1 Department of Obstetrics and Gynecology, Peking University First Hospital, No. 8 Xishiku Street, Beijing, 100034 China; 2 Beijing Key Laboratory of Maternal Fetal Medicine of Gestational Diabetes Mellitus, Beijing, 100034 China

**Keywords:** Hyperglycemia in pregnancy, Gestational diabetes mellitus, Galectin-3, Apoptosis

## Abstract

**Objective:**

This study was to evaluate plasma galectin-3 levels from early pregnancy to delivery and explore the effects of galectin-3 on the function of trophoblast cells under high glucose exposure.

**Methods:**

The plasma galectin-3 levels were quantified by enzyme-linked immunosorbent assay (ELISA) in the China National Birth Cohort (CNBC) at Peking University First Hospital, and the underlying signaling pathway was identified by protein–protein interaction (PPI) analysis, gene set enrichment analysis (GSEA), quantitative PCR (qPCR), western blotting, small interfering RNA (siRNA) transfections, and flow cytometry.

**Results:**

Significantly higher galectin-3 levels were found in patients with gestational diabetes mellitus (GDM group; *n* = 77) during the first and second trimesters than that in healthy pregnant women (HP group; *n* = 113) (*P* < 0.05). No significant differences in plasma galectin-3 levels were detected between GDM and HP groups in maternal third-trimester blood and cord blood. PPI analysis suggested potential interactions between galectin-3 and foxc1. The findings of GSEA showed that galectin-3 was involved in the cytochrome P450-related and complement-related pathways, and foxc1 was associated with type I diabetes mellitus. Additionally, high glucose (25 mM) significantly increased the expression levels of galectin-3 and foxc1 and induced apoptosis in HTR-8/SVneo cells. Further in vitro experiments showed that galectin-3/foxc1 pathway could protect HTR-8/SVneo cells against high glucose − induced apoptosis.

**Conclusion:**

Future studies were required to validate whether plasma galectin-3 might become a potential biomarker for hyperglycemia during pregnancy. Elevated galectin-3 levels might be a vital protective mechanism among those exposed to hyperglycemia during pregnancy.

**Graphical Abstract:**

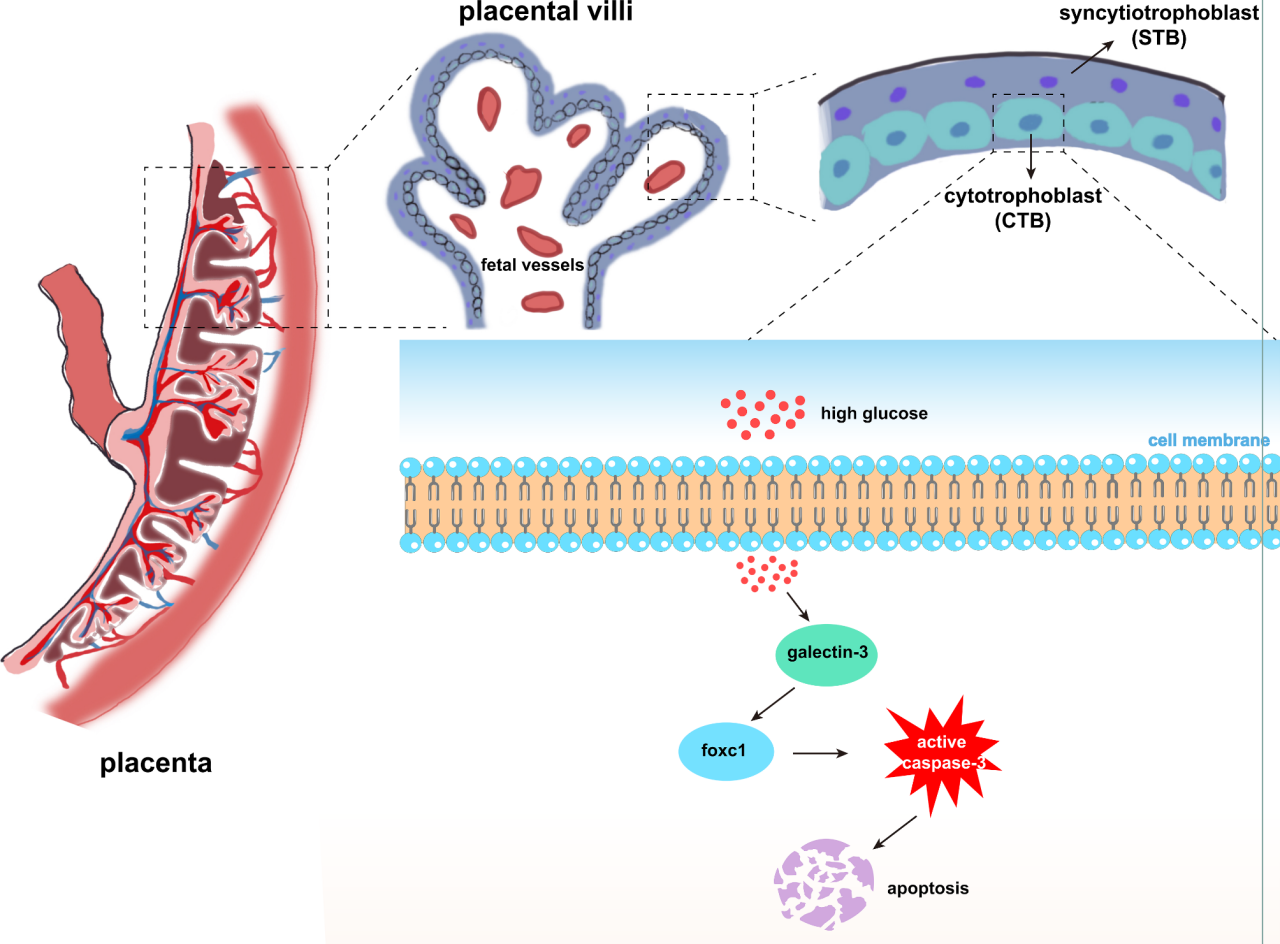

**Supplementary Information:**

The online version contains supplementary material available at 10.1186/s10020-023-00707-5.

## Introduction

Hyperglycemia was the most common complication in pregnancy, estimated to affect 16.8% of pregnancies worldwide (Hod et al. 2015). Hyperglycemia in pregnancy (HIP) increased risks and complications of short-term and long-term. For short-term effect, hyperglycemia and adverse pregnancy outcome (HAPO) study (*n* = 25 505; including 15 centers) reported that continuous and graded relationship between maternal hyperglycemia and the risk of adverse outcomes (including premature delivery, shoulder dystocia or birth injury, intensive neonatal care, hyperbilirubinemia, and preeclampsia) (Metzger et al. 2008). For long-term effect, HAPO Follow Up Study (HAPO-FUS) (*n* = 4 834; including 10 centers) showed that HIP was significantly related with glucose and insulin resistance in children, independent of maternal and child body mass index (BMI) and family history of diabetes (Scholtens et al. 2019). Therefore, it was of great significance for controlling blood glucose during pregnancy to reduce the risk of short- and long-term adverse pregnancy outcomes.

Previous studies (Sweeting et al. 2016; Geurtsen et al. 2019) had shown that hyperglycemia in early pregnancy was associated with adverse pregnancy outcomes, these findings provided some insights into earlier interventions of GDM. Even with intensive interventions for early GDM (diagnosed at < 12 weeks of gestation), early GDM had significantly higher incidence of adverse pregnancy outcomes than later GDM (diagnosed after 24 weeks of gestation), such as macrosomia (20.3% vs. 10.0%), jaundice (28.1% vs. 19.8%), and respiratory distress syndrome (7.4% vs. 4.0%) (Sweeting et al. 2016). The risk of delivering infants of large for gestational age increased with per 1 mmol/L increase in non-fasting glucose levels in early pregnancy (odds ratios [ORs], 1.21; 95% CI, 1.10–1.34) (Geurtsen et al. 2019). Therefore, early identification and intervention for HIP had important clinical implications.

Galectin-3 (*LGALS3*) was a member of the β-galactoside-binding lectins family that widely regulated intercellular and extracellular interactions in biological organism, and mainly localized in cytoplasm region, but could be also seen in the perinuclear membrane and nucleus (Li et al. 2022). Several studies (Yilmaz et al. 2015; Mensah-Brown et al. 2009; Li et al. 2016; Pejnovic et al. 2013; Darrow and Shohet 2015) suggested that hyperglycemia was linked to galectin-3. Yilmaz et al. (2015) showed serum levels of galectin-3 were significantly elevated in type 2 diabetes mellitus and prediabetic patients compared with healthy controls, and were positively correlated with fasting plasma glucose (*r* = 0.787, *P* < 0.01) and 2-h plasma glucose levels (*r* = 0.833, *P* < 0.01). In addition, galectin-3 also played influential roles in the development of diabetes. Mensah-Brown et al. (2009) found that galectin-3 knock-out (KO) mice displayed markedly reduced blood glucose levels compared to wide-type (WT) mice after the treatment of multiple low dose streptozotocin. Li et al. (2016) exhibited that galectin-3 KO mice were much more glucose-tolerant and had significantly lower basal insulin levels than WT mice, after high-fat feeding. Mechanistically, galectin-3 inhibited key steps in the insulin signaling pathway, including phosphorylation of insulin receptor, phosphoinositide-dependent protein kinase 1 and Akt (Li et al. 2016). However, studies by Pejnovic et al. (2013) and Darrow AL et al. (Darrow and Shohet 2015) showed different results. Pejnovic et al. (2013) presented that fasting blood glucose levels, fasting insulin levels and glycohemoglobin for galectin-3 KO mice were significantly higher than WT mice, when fed a high-fat diet. Darrow AL et al. (Darrow and Shohet 2015) found that galectin-3 KO mice with high-fat diet displayed worse glucose tolerance and higher fasting glucose levels than WT mice with high-fat diet. Therefore, further studies about galectin-3 were needed to explain the differences in these results.

Galectin-3 played many physiologic and pathologic roles in pregnancy-associated diseases including gestational diabetes mellitus (Talmor-Barkan et al. 2020; Freitag et al. 2020a; Heusler et al. 2021; Wang et al. 2022), fetal growth restriction (Hutter et al. 2016; Freitag et al. 2020b), infertility (Yang et al. 2016), and missed abortion (Xiao et al. 2019). Talmor-Barkan et al. (2020) implied that serum galectin-3 levels of gestational diabetes mellitus (GDM) group were significantly higher than that in normal pregnant women during the first and third trimester in Israeli population. Recent study (Tang and Chen 2022) showed galectin-3 levels in the blood of GDM group significantly related to adverse pregnancy outcomes (*r* = 0.698, *P* < 0.05). Furthermore, Freitag et al. (2020a) observed that galectin-3 could promote cell fusion and tube formation capacity of trophoblast cells. However, to the best of our knowledge, there had been no reports of plasma galectin-3 levels from early pregnancy to delivery in Chinese Han populations. Noteworthy, the placenta was an extremely important endocrine organ during pregnancy, which significantly influenced the health of the fetus by providing material exchange (Knöfler et al. 2019). The effect of galectin-3 on placental trophoblast cell function under high glucose exposure had not been fully studied. Thus, we aimed to examine plasma galectin-3 levels from early pregnancy to delivery in the same cohort, and explore the effect of galectin-3 on placental trophoblast cells under high glucose exposure for gaining a detailed understanding of HIP.

## Materials and methods

### Population

The population of this study was from a sub-cohort (between July 2017 and December 2020) of the China National Birth Cohort (CNBC) at Peking University First Hospital. CNBC was a multicenter prospective birth cohort study funded by the Ministry of Science and Technology of China, and the cohort methodology and study design had been previously described in detail (Hu et al. 2021; Jiang et al. 2021). The present study included 113 healthy pregnant women (HP) (mean age, 31.48 years) and 77 patients with GDM (mean age, 32.97 years). Study inclusion criteria were as follows: (i) pregnancy women between 8 and 14 weeks of gestation; (ii) natural conception; (iii) plan to have routine prenatal checkups and delivery in Peking University First Hospital. HP group was defined as pregnant women without complications. All participants were entirely voluntary, and each participant signed an informed consent form. The Ethics Committee of the Peking University First Hospital approved this study prior to the commencement of research. Previous study (Hellmuth et al. 2017) had shown that pre-pregnancy BMI had a greater impact on maternal metabolic adaptation than weight gain or dietary intake during pregnancy, highlighting the importance of pre-pregnancy BMI. The analysis of pre-pregnancy BMI was not negligible in pregnancy-related studies (Page et al. 2019). Thus, maternal pre-pregnancy BMI (kg/m^2^) was classified into two groups: the normal weight group (pre-pregnancy BMI ≥ 18.5 and < 24.0 kg/m^2^) and overweight or obesity group (pre-pregnancy BMI ≥ 24.0 kg/m^2^) by the Chinese BMI standards (Zhou 2002).

### Plasma sample preparation and measurement

Blood samples were collected from the pregnancy women at the first trimester (< 13 weeks), second trimester (≥ 13 weeks to < 27 weeks) and delivery. Maternal blood was collected during pregnancy and cord blood was collected from the umbilical vein. Subsequently, blood samples were centrifuged at 1,500 x g (room temperature) for 15 min to prepare plasma samples, and plasma samples were stored at − 80 °C until biochemical measurements. Plasma galectin-3 concentrations were measured by ELISA (#ml028578, Mlbio, Shanghai, China), according to the manufacturer’s protocol. To reduce the error, the ELISA test was completed by the experimenter who had at least five years of professional experiences. ELISA plates were washed with an automatic plate washer (#5165000, Thermo Fisher Scientific, Shanghai, China) and absorbance was measured using spectrophotometry (Thermo Fisher Scientific, Vantaa, Finland).

### Diagnosis of GDM

The oral glucose tolerance test (OGTT) was conducted in pregnant women between 24 and 28 weeks of gestation, following the international association of diabetes and pregnancy study groups (IADPSG) recommendations (Metzger et al. 2010). The diagnosis of GDM was made if one or more of the following criteria were met: fasting blood glucose > 5.1 mmol/L, blood glucose at 1 h after loading glucose > 10.0 mmol/L, blood glucose at 2 h after glucose intake > 8.5 mmol/L (Metzger et al. 2010).

### Protein–protein interaction (PPI) analysis

PPI networks were generated using GeneMANIA (https://genemania.org/) (Franz et al. 2018) to analyze proteins or genes interacted or co-expressed with *LGALS3*. The interaction networks included physical interactions, co-expression, co-localization, genetic interactions, shared protein domains and pathways.

### Gene set enrichment analysis (GSEA)

Gene expression profiles of human placental samples that contained both GDM (*n* = 4) and HP (*n* = 4) groups were downloaded from the Gene Expression Omnibus (GEO) database: GSE154414 (Wang et al. 2021a). Overall, there were no statistically significant differences in baseline characteristics (such as maternal age, gestational age, prepregnancy BMI, and newborn birth weight) between GDM and HP groups. For further demographic information about these samples were reported in previous study (Wang et al. 2021a).

For further analysis, we converted fragments per kilobase of exon model per million mapped fragments (FPKM) to transcripts per kilobase million (TPM) before normalization (Dalal et al. 2022). And then, to determine the effect of *LGALS3* on placenta, we divided the samples into *LGALS3* high-expression and *LGALS3* low-expression groups according to the median value of *LGALS3* expression, followed using GSEA (Mao et al. 2022). For GSEA, the screening condition for significantly enriched pathways was adj *P*.value < 0.05.

### Immunohistochemistry (IHC)

IHC was performed according to standard protocol as described by previous study (Jenkins et al. 2018). Briefly, human placenta tissues were fixed and embedded in paraffin, and cut into 5 μm sections before deparaffinization and antigen retrieval. Then, sections were incubated with primary antibodies anti-galectin-3 (#ab2785, Abcam; dilution rates of 1:200) at 4 °C overnight. After washing with PBS, slides were incubated with secondary antibodies for 60 min at 37 °C. Finally, the slides were stained with diaminobenzidine (DAB, #ZLI-9017, ZSGB-Biotech, Beijing, China) and counterstained with haematoxylin. Five visual fields (×40) were randomly chosen per each sample (*n* = 4), and imaged for analysis using the Image Pro Plus software. Mean density = IOD/area.

### Reagents

D-glucose (#G8150, Solarbio, Beijing, China) was dissolved in normal medium until reached a targeted glucose concentration. Recombinant Human Galectin-3 (#DC068) was purchased from Novoprotein (Shanghai, China).

### Cell culture and treatment with high glucose

HTR-8/SVneo cells were cultured in PRMI-1640 (#MA0215, Meilunbio, China) and supplemented with 10% fetal bovine serum (FBS; Biological Industries, Beit-Haemek, Israel) and 1% penicillin-streptomycin. When cells used for experiments, cells were cultured in medium containing 5 mmol/L glucose for 24 h, and then cultured in media containing 5 mmol/L, 11 mmol/L, and 25 mmol/L glucose (representing hyperglycemia) for 48 h, respectively (Hahn et al. 2000). Cells were incubated in humidified air at 37 °C in a 5% CO2 incubator.

### RNA extraction and quantitative polymerase chain reaction (qPCR)

For analysis of gene expression, the total cellular RNA was extracted using the TRIzol reagent (#15596018, Ambion, Austin, TX, USA), according to the manufacturer’s instructions. cDNA synthesis was performed using the FastKing gDNA Dispelling RT SuperMix (#KR118-02, TIANGEN Biotech). qPCR was carried out using Power 2x SYBR Green qPCR Master Mix (#B21702, Bimake) using ABI Prism 7500 platform (Applied Biosystems, Singapore). The forward and reverse primers used were listed in Supplementary Table 1. The relative gene expression was calculated using the 2 − ΔΔCT method after normalizing to GAPDH expression.

### Western blotting

Cells were lysed in lysis buffer (#KGP250/KGP2100, Keygen Biotech, Nanjing, China), and proteins were separated by SDS–polyacrylamide gel electrophoresis (SDS–PAGE) before transferring to PVDF membranes, according to the standard manufacture’s protocol. Vinculin (Gudiño et al. 2021) and β-Actin were used as internal loading control. The following primary antibodies were used in this study: galectin-3 (#ab2785, Abcam; dilution rates of 1:1000), foxc1 (#ab227977, Abcam; dilution rates of 1:1000), active caspase-3/caspase-3 (#A11021 and #A19654, ABclonal, Wuhan, China; dilution rates of 1:500), and vinculin (#26520-1-AP, Proteintech, China; dilution rates of 1:2000). All primary antibodies were incubated with PVDF membranes overnight at 4 °C. The incubation time for active caspase-3/caspase-3 primary antibody needed to be appropriately longer. Images of protein bands were captured using Syngene GeneGenius (SYNGENE, GeneGnome XRQ NPC, Cambridge, UK.).

### Knockdown analysis using siRNAs

The galectin3 small interfering RNA (siRNA), foxc1 siRNA and scrambled sequence siRNA (negative control siRNA, NC-siRNA) were purchased from GenePharma (GenePharma Co., Ltd., Shanghai, China). The specific sequence for galectin3 siRNA was 5’- CCAUCUUCUGGACAGCCAATT-3’ (sense) and 5’-UUGGCUGUCCAGAAGAUGGTT-3’ (antisense). Two siRNAs for foxc1 designed and synthesized were as follows: foxc1-siRNA-516: 5’-GCGCUUCAAGAAGAAGGACTT-3’ (sense) and 5’- GUCCUUCUUCUUGAAGCGCTT-3’ (antisense); and foxc1-siRNA-1409: 5’-UCACCUCGUGGUACCUGAATT-3’ (sense) and 5’- UUCAGGUACCACGAGGUGATT-3’ (antisense). Briefly, siRNA transfections were performed with 50 nM siRNA duplexes using RNAiMAX reagent (Invitrogen), according to the manufacturer’s instructions.

### Flow cytometry of the cell apoptosis

Cells were cultivated in a six-well plate (2 × 10^5^ cells/well). After treatment, cells were harvested and cell apoptosis was performed using Annexin V-FITC/PI apoptosis kit (#AT101, MultiSicences, China) according to the manufacturer’s protocol. Flow cytometric analysis were conducted on a BD LSR Fortessa X-20 cell analyzer (BD Biosciences, USA).

### Statistical analysis

Baseline tables and Spearman correlation analysis were completed using R statistical software (R version 4.1.2). The data presented in the figure were statistically analyzed with GraphPad Prism 8 software (GraphPad Software, v8.0.1, San Diego, USA). ELISA, qPCR, western blotting and flow cytometry data were analyzed using two-tailed unpaired Student’s T test. *P* < 0.05 was considered significant (*), *P* < 0.01 was considered highly significant (**), and *P* < 0.001 was considered very highly significant (***).

## Results

### Study participants’ characteristics

The demographic and clinical baseline characteristics were shown in Table 1. This study included a total of 190 individuals; of those, 113 participants were healthy pregnant women and the remaining 77 participants had GDM. The overall mean maternal age and gestational week of delivery were 32.07 ± 3.85 years and 39.19 ± 0.99 weeks, respectively. The GDM group had a higher pre-pregnancy BMI (23.80 ± 3.53 vs. 21.26 ± 2.26 kg/m^2^; *P* < 0.001), compared to the HP group. The GDM group presented higher OGTT-0 min (5.38 ± 0.27 vs. 4.47 ± 0.21 mmol/L), OGTT-60 min (9.47 ± 1.74 vs. 7.34 ± 1.27 mmol/L), OGTT-120 min (8.19 ± 1.57 vs. 6.25 ± 1.11 mmol/L) values than the HP group, with significant differences between the groups (*P* < 0.001).

**Table 1:** Baseline characteristics of the study population

	Level	GDM	HP	*P*-value	Statistics
***N***		77	113		
**Maternal_age** **(Mean (SD))**		32.97 (3.85)	31.48 (3.75)	0.009	t test
**Gestational_week_** **of_delivery** **(Mean (SD))**		38.95 (0.92)	39.35 (1.01)	0.006	t test
**Gravidity (%)**				0.213	Chi-square test
	1	20	23		
	2	31	54		
	3	13	26		
	≥ 4	13	10		
**Parity (%)**				0.342	Chi-square test
	1	46	75		
	2	30	38		
	≥ 3	1	0		
**Prepregnancy_weight** **(Mean (SD))**		64.49 (11.86)	56.67 (8.03)	< 0.001	t test
**Prepregnancy_BMI** **(Mean (SD))**		23.80 (3.53)	21.26 (2.26)	< 0.001	t test
**Weight_gain** **(Mean (SD))**		11.76 (4.19)	13.09 (3.95)	0.032	t test
**Mode_of_delivery (%)**				< 0.001	Fisher’s exact test
**Cesarean**		41	31		
**Spontaneous**		36	82		
**Placental_weight** **(Mean)**		584.17	567.80	0.106	t test
**OGTT_0** **(Mean (SD))**		5.38 (0.27)	4.47 (0.21)	< 0.001	t test
**OGTT_60** **(Mean (SD))**		9.47 (1.74)	7.34 (1.27)	< 0.001	t test
**OGTT_120** **(Mean (SD))**		8.19 (1.57)	6.25 (1.11)	< 0.001	t test

Abbreviations: GDM, gestational diabetes mellitus; HP, healthy pregnant women; OGTT, oral glucose tolerance test

### Comparison of plasma galectin-3 levels between GDM and HP groups

**Fig. 1:**
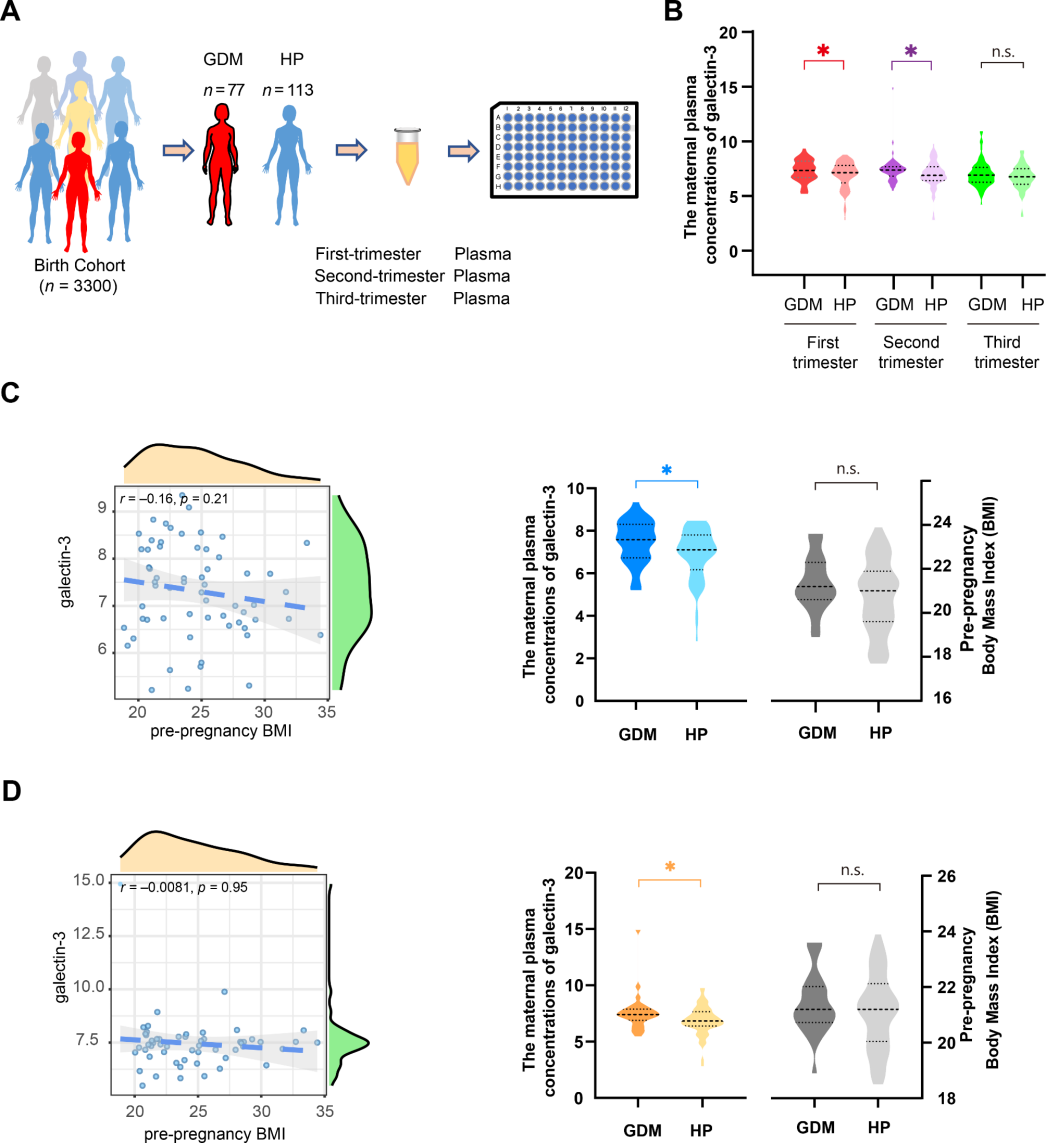
The plasma galectin-3 levels during pregnancy. **(A)** Flowchart of the study for detecting plasma galectin-3 levels by using enzyme-linked immunosorbent assay (ELISA). **(B)** Plasma galectin-3 levels in gestational diabetes mellitus (GDM) and healthy pregnant women (HP) groups during pregnancy. **(C-D)** Plasma galectin-3 levels between GDM with the normal weight group (pre-pregnancy BMI ≥ 18.5 and < 24.0 kg/m2) and HP with the normal weight group (pre-pregnancy BMI ≥ 18.5 and < 24.0 kg/m2) during the first (C) and second (D) trimesters. Correlation between plasma galectin-3 levels and pre-pregnancy BMI (left panel). GDM with the normal weight group had significant higher maternal plasma galectin-3 levels than HP with the normal weight group (middle panel). The GDM with the normal weight group and HP with the normal weight group had similar pre-pregnancy BMI (right panel). n.s., no significant difference. *: *P* < 0.05, **: *P* < 0.01

To further investigate the plasma galectin-3 levels during pregnancy (especially in the first and second trimesters), ELISA assays were performed in GDM and HP groups (Fig. 1A). The correlation coefficients (R 2) for each standard curve were above 0.99. The results showed the GDM group had higher maternal plasma galectin-3 levels compared with the HP group (7.29 vs. 6.90 ng/mL; *P* = 0.041) during the first trimester (Fig. 1B). The GDM group showed higher maternal plasma galectin-3 levels (7.41 vs. 6.95 ng/mL; *P* = 0.036) than the HP group in the second trimester (Fig. 1B). No significant statistical differences of plasma galectin-3 levels were observed between GDM and HP groups in third trimester (Fig. 1B) and cord blood (Supplementary Fig. 1).

To further evaluate the association between plasma galectin-3 levels and pre-pregnancy BMI, Spearman correlation test and subgroup analysis were conducted. We first analyzed the correlation between plasma galectin-3 levels and pre-pregnancy BMI, showing a low correlation in the first (*r* = − 0.16, *P* = 0.21; Fig. 1C) and second trimesters (*r* = − 0.0081, *P* = 0.95; Fig. 1D), without statistical significance. Next, GDM and HP groups were subdivided into the normal weight group (pre-pregnancy BMI ≥ 18.5 and < 24.0 kg/m^2^) and overweight or obesity group (pre-pregnancy BMI ≥ 24.0 kg/m^2^), respectively, according to the Chinese BMI standards (Zhou 2002). GDM with the normal weight group had higher plasma galectin-3 levels (the first trimester: 7.47 vs. 6.89 ng/mL; *P* = 0.021 and second trimester: 7.61 vs. 6.88 ng/mL; *P* = 0.027) than the HP with the normal weight group, but there were no statistically significant differences in pre-pregnancy BMI between the two groups (Fig. 1C and D).

### High glucose increased the expression levels of galectin-3 and foxc1

**Fig. 2:**
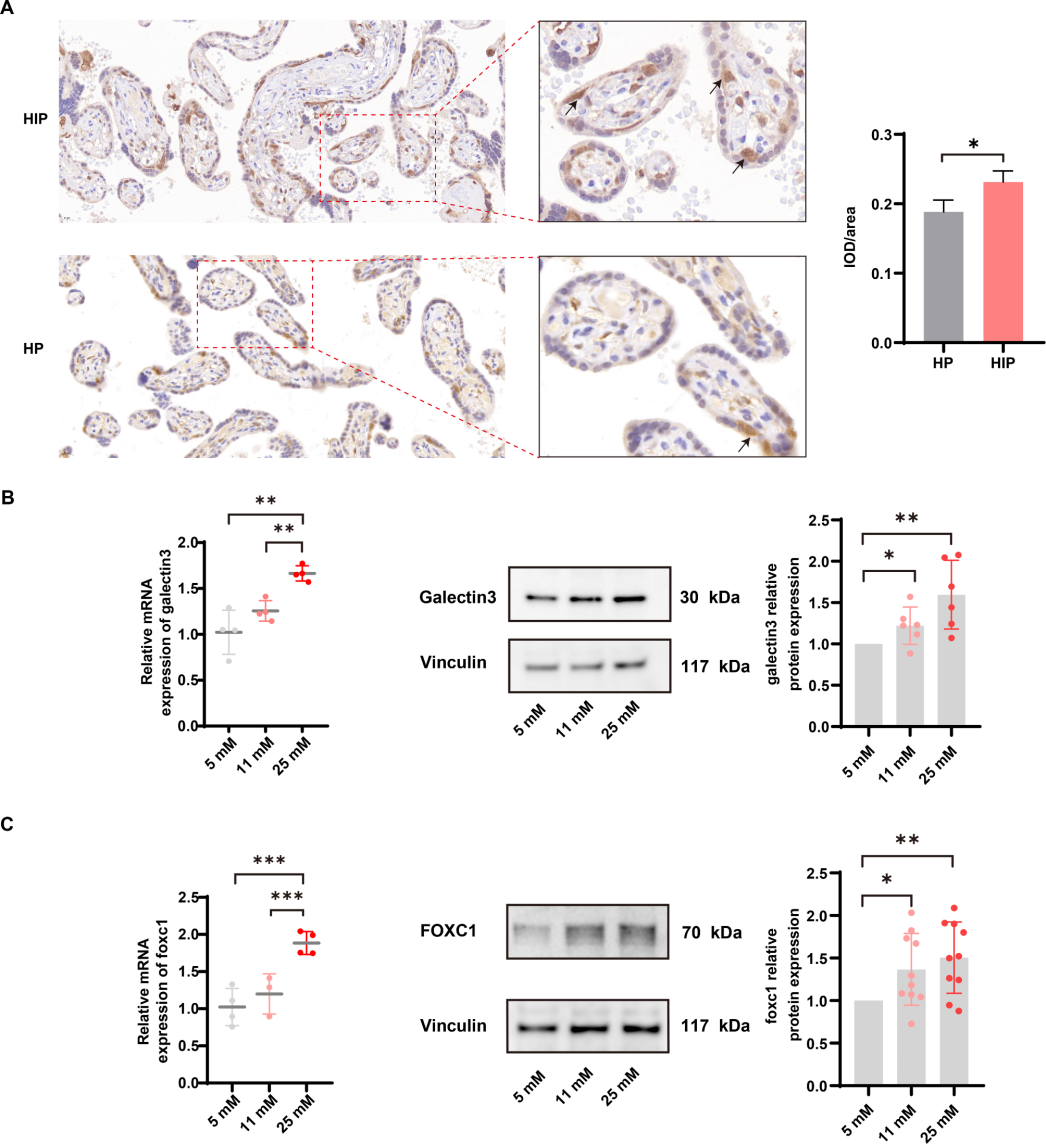
Effects of different glucose concentrations on the expression levels of galectin-3 and foxc1. **(A)** Expression of galectin-3 in human placenta of hyperglycemia in pregnancy (HIP, *n* = 4) and healthy pregnant women (HP, *n* = 4). Abundant expression of galectin-3 protein observed in cytotrophoblast (CTB) (arrows). **(B)** Relative mRNA expression levels of galectin-3 determined by RT-qPCR in HTR-8/SVneo cells (left panel). Expression of galectin-3 protein levels measured by western blotting in HTR-8/SVneo cells (middle and right panels). **(C)** Relative mRNA expression levels of foxc1 determined by RT-qPCR in HTR-8/SVneo cells (left panel). Expression of foxc1 protein levels measured by western blotting in HTR-8/SVneo cells (middle and right panels). *: *P* < 0.05, **: *P* < 0.01, and ***: *P* < 0.001

To confirm localization of galectin-3 in placental tissues, we conducted immunohistochemistry. We found that galectin-3 protein expression was negative in syncytiotrophoblast (STB), where galectin-3 protein expression was observed abundant (arrows) in cytotrophoblast (CTB) (Fig. 2A). To explore the expression levels of galectin-3 and foxc1 in high glucose conditions, we cultured the HTR-8/SVneo cells for 48 h in normal glucose medium (5mM), moderate glucose medium (11 mM) and high glucose medium (25 mM), respectively. Both qPCR and western blotting results indicated that the expression levels of galectin-3 and foxc1 increased with the higher glucose concentration (Fig. 2B C).

### The expression of foxc1 was regulated by galectin-3

**Fig. 3:**
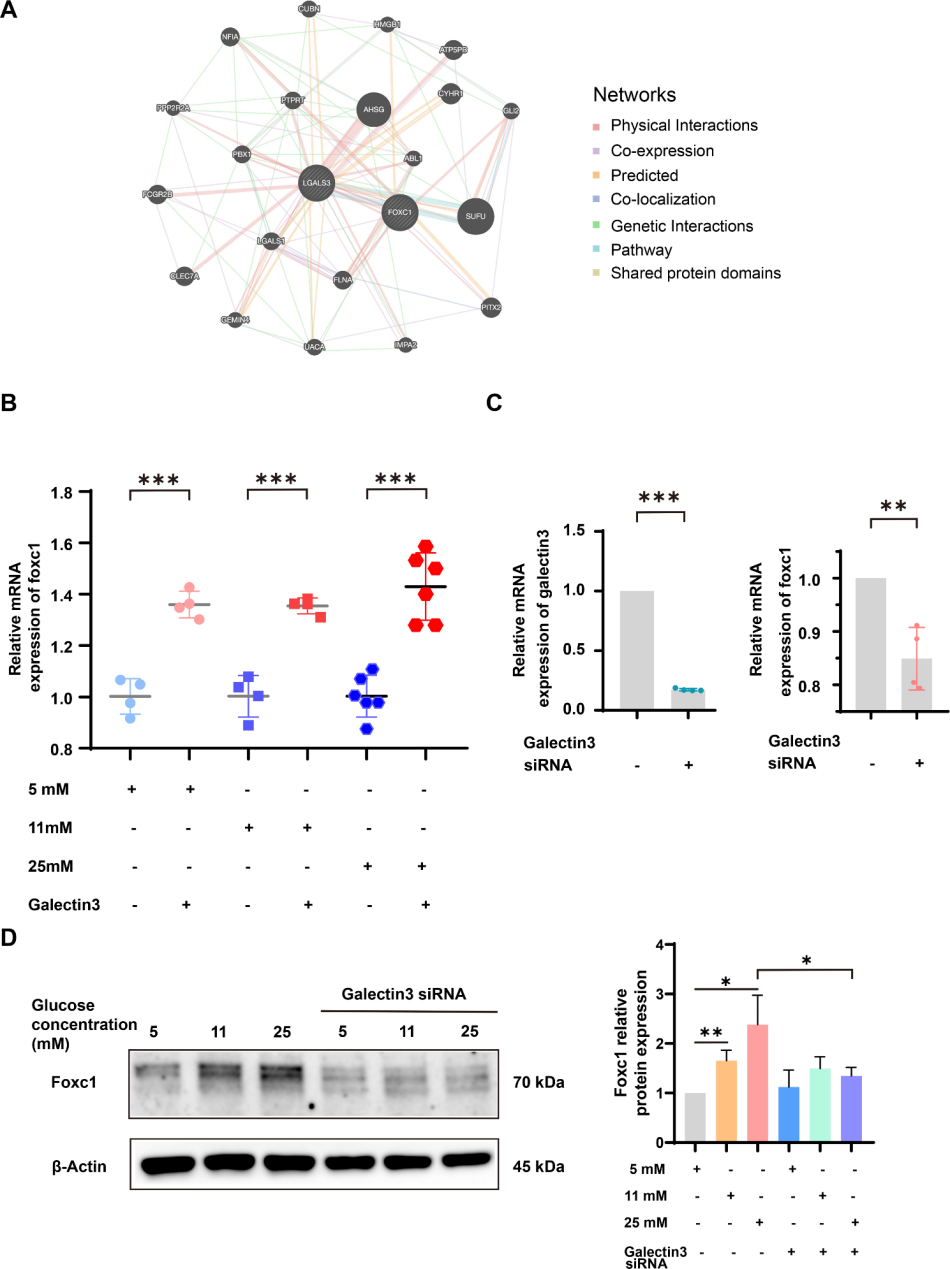
The regulation of foxc1 expression by galectin-3 in human HTR-8/SVneo cells. **(A)** PPI network of galectin-3 (*LGALS3*) and foxc1 was constructed by GeneMANIA.The interconnections between galectin-3 (*LGALS3*) and foxc1 were explored in term of physical interactions, co-expression, co-localization, genetic interactions, shared protein domains and pathways. **(B)** Recombinant human galectin-3 up-regulated the mRNA expression level of foxc1. **(C)** The relative mRNA expression levels of galectin-3 (left panel) and foxc1 (right panel) in HTR-8/SVneo cells transfected with galectin-3 siRNA. **(D)** Western blotting analysis and quantification of the expression of foxc1 protein level in HTR-8/SVneo cells treated with galectin-3 siRNA and different glucose concentration (5 mM, 11 mM, and 25 mM). *: *P* < 0.05, **: *P* < 0.01, and ***: *P* < 0.001

In our team’s previous single cell analysis results, we found that foxc1 expression was significantly increased in the placenta of patients with high glucose exposure during pregnancy (data not shown). The PPI network analysis revealed complex interactions between galectin-3 and foxc1 (Fig. 3A). Because of that, we explored whether galectin-3 regulated foxc1 expression. Results showed that the gene expression of foxc1 was remained at a low level, whereas its gene expression significantly increased after the addition of recombinant galectin3 (Fig. 3B). To further validate the regulation of galectin-3 on foxc1, we negatively regulated galectin3 expression with siRNA (Fig. 3C). qPCR results indicated that the gene expression of foxc1 was significantly decreased after galectin-3 knockdown (*P* < 0.01) (Fig. 3C). Consistent with the qPCR results, the western blotting results showed foxc1 was regulated by galectin3 in cells exposed to high glucose (25 mM) (*P* < 0.05) (Fig. 3D).

### Galectin-3/foxc1 pathway negatively regulated apoptosis in HTR-8/SVneo cells

**Fig. 4:**
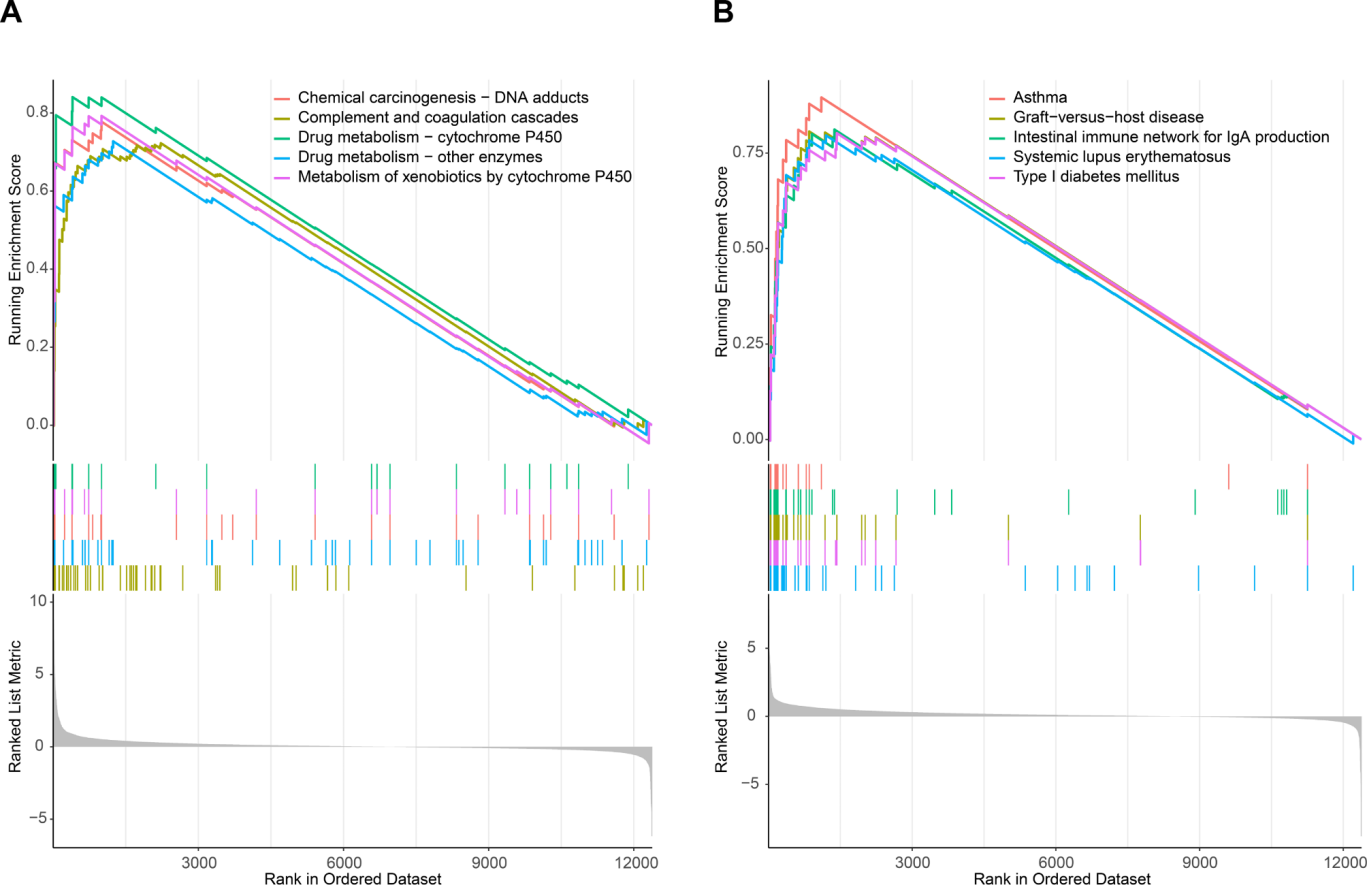
Gene set enrichment analysis (GSEA) of the galectin-3 and foxc1 in the GSE154414 dataset. **(A)** The top 5 pathways enriched in the high galectin-3 expression group according to GSEA enrichment score. **(B)** The top 5 pathways enriched in the high foxc1 expression group according to GSEA enrichment score

**Fig. 5:**
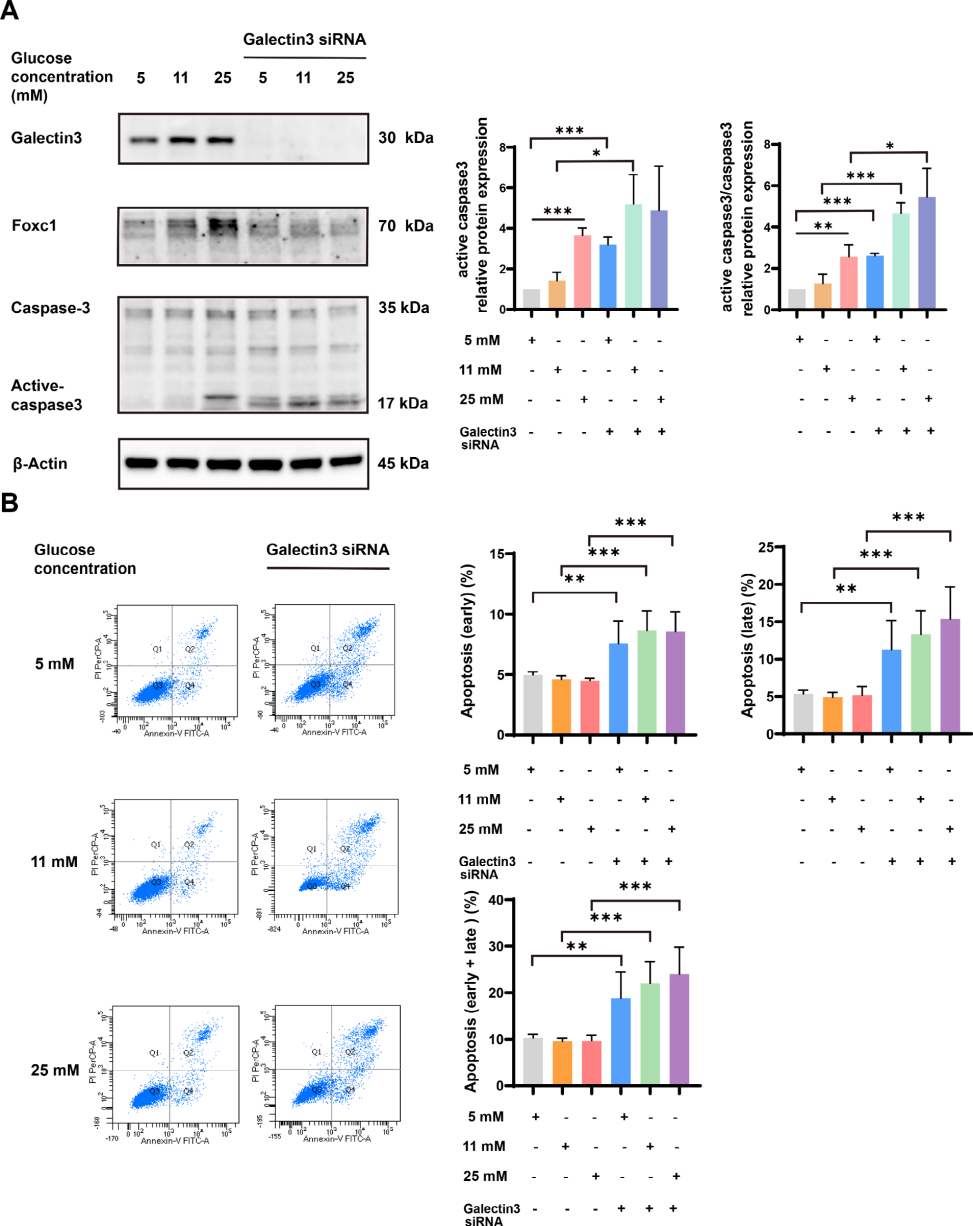
Galectin-3 antagonized high glucose (25 mM) − induced trophoblast cells apoptosis. **(A)** Western blotting analysis showing expression of pro-apoptosis markers (active caspase-3 and total caspase-3) with galectin-3 siRNA. **(B)** The percentages of apoptosis (early apoptosis, late apoptosis, and early apoptosis plus late apoptosis) of HTR-8/SVneo cells were measured by flow cytometry after treatment with galectin-3 siRNA. Q1, necrotic cells; Q2, late apoptosis cells; Q3, living cells; Q4, early apoptosis cells. *: *P* < 0.05, **: *P* < 0.01, and ***: *P* < 0.001

To further explore the potential biological role of galectin-3 in placenta, GSEA was performed based on the placental RNA sequencing data from GSE154414. The GSEA results showed that the galectin-3 was primarily associated with drug metabolism − cytochrome P450, metabolism of xenobiotics by cytochrome P450, and complement and coagulation cascades signaling pathways (Fig. 4A). GSEA results further showed a positive correlation between foxc1 and type I diabetes mellitus (Fig. 4B). Previous study showed the P450 enzymes were closely related to apoptosis (Hua et al. 2013). Therefore, we speculated that galectin-3 was related to apoptosis. To test the hypothesis, protein expression levels of the apoptosis-related markers (active caspase-3 and total caspase-3 (Wei et al. 2021) were detected by western blotting (Fig. 5A). Western blotting results indicated that galectin-3 knockdown significantly increased the protein expression levels of reliable pro-apoptosis markers (active caspase-3 and total caspase-3), and significantly induced caspase-3 cleavage to form cleaved caspase-3 (active caspase-3), suggesting a negative regulation of cell apoptosis by galcectin3 (Fig. 5A). Furthermore, flow cytometry analysis was performed to confirm the results obtained by western blotting (Fig. 5B). Flow cytometry results revealed that knocking down galectin-3 promoted apoptosis (including early apoptosis, late apoptosis, and early apoptosis plus late apoptosis) of HTR-8/SVneo cells exposed to high glucose (25 mM) (*P* < 0.05), which were consistent with the results of western blotting (Fig. 5B).

**Fig. 6:**
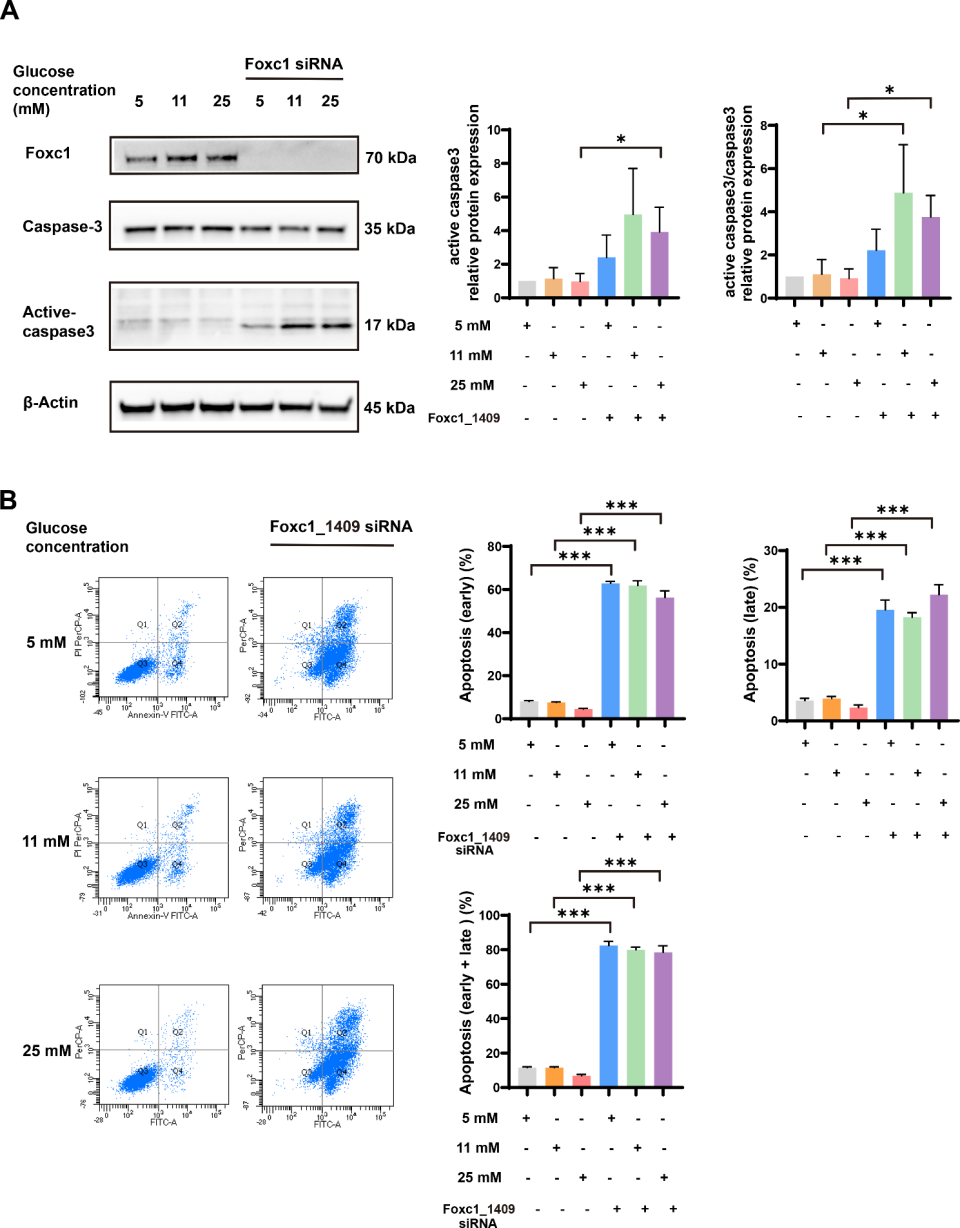
Foxc1 antagonized high glucose (25 mM) − induced trophoblast cells apoptosis. **(A)** Western blotting analysis showing expression of pro-apoptosis markers (active caspase-3 and total caspase-3) with foxc1 siRNA. **(B)** The percentages of apoptosis (early apoptosis, late apoptosis, and early apoptosis plus late apoptosis) of HTR-8/SVneo cells were measured by flow cytometry after treatment with foxc1-siRNA-1409. Q1, necrotic cells; Q2, late apoptosis cells; Q3, living cells; Q4, early apoptosis cells. *: *P* < 0.05, **: *P* < 0.01, and ***: *P* < 0.001

To further explore the role of galectin-3/foxc1 pathway in HTR-8/SVneo cells, two different siRNA-mediated foxc1 knockdown was employed (foxc1-siRNA-1409 and foxc1-siRNA-516). Knockdown efficiencies of foxc1-siRNA-1409 and foxc1-siRNA-516 were 73% and 79%, respectively (Supplementary Fig. 2). Western blotting showed that the expression of apoptotic proteins (active caspase-3) and the apoptotic ratio of active caspase-3/total caspase-3 was significantly increased after foxc1 knockdown, suggesting a negative regulation of cell apoptosis by foxc1 (Fig. 6A). Similar to our previous results (Fig. 6A), the proportion of early apoptosis, late apoptosis, and early apoptosis plus late apoptosis was markedly higher in foxc1-siRNA-1409 treatment group than the negative control siRNA group (*P* < 0.05) (Fig. 6B). Moreover, another siRNA against foxc1 (foxc1-siRNA-516) showed the similar result as did foxc1-siRNA-1409 (Supplementary Fig. 3). Taken together, these results revealed that cell apoptosis was negatively regulated by galectin-3/foxc1 pathway.

## Discussion

This study presented evidence that GDM patients with significantly higher maternal plasma galectin-3 levels than healthy pregnant women during the first and second trimesters. However, no significant differences in maternal plasma galectin-3 levels between GDM patients and healthy pregnant women were observed during the third trimesters. Based on PPI and GSEA analysis, we predicted and identified previously unreported galectin-3/foxc1 pathway. Our findings suggested galectin-3/foxc1 pathway protected HTR-8/SVneo cells against high glucose − induced apoptosis, which might be related to the important function of galectin-3 in maintaining fetal development (Freitag et al. 2020b).

Recent studies suggested that serum galectin-3 might be a potential biomarker for polycystic ovary syndrome (Anik Ilhan et al. 2018), poststroke cognitive impairment (Wang et al. 2021b) and Idiopathic inflammatory myopathies complicated by interstitial lung disease (Watanabe et al. 2021). Previous research had indicated serum galectin-3 might serve as a predictive biomarker for GDM in first trimester, with satisfactory accuracy (area under the curve [AUC] = 0.734) (Talmor-Barkan et al. 2020). Given that investigations (Boutsikou et al. 2015) on the plasma levels of galectin-3 at different gestational periods (from early pregnancy to delivery) were limited, we found that the GDM group had higher plasma galectin-3 levels than the HP group during the first and second trimester in this present study. However, there was no significant difference of plasma galectin-3 levels between GDM and HP groups in maternal blood and cord blood, which might be associated with satisfactory glucose control in both groups during late pregnancy (data not shown).

In a previous study (Weigert et al. 2010), serum galectin-3 level was positively associated with BMI in type 2 diabetes mellitus patients (*r* = 0.357, *P* = 0.001). Yilmaz et al. (2015) reported that there were no correlations between galectin-3 and BMI in the whole population (*n* = 174). In our study, we observed a low correlation of plasma galectin-3 level and pre-pregnancy BMI in the first (*r* = − 0.16, *P* = 0.21) and second trimesters (*r* = − 0.0081, *P* = 0.95). In addition, we performed subgroup analysis to investigate whether the plasma galectin-3 levels depended on pre-pregnancy BMI. The subgroup analysis showed GDM patients had significantly higher plasma galectin-3 levels than the HP in the first and second trimesters (*P* < 0.05) in the normal weight group (pre-pregnancy BMI ≥ 18.5 and < 24.0 kg/m^2^), but no statistically significant differences were found in the pre-pregnancy BMI between GDM and HP groups. Collectively, these results suggested that galectin-3 might be a potential candidate biomarker for GDM, and further investigation was required.

Galectin-3 played important roles in apoptosis (Saksida et al. 2013), cellular autophagy (Burbidge et al. 2022), and cell invasion (Freitag et al. 2020a; Serizawa et al. 2015). However, the roles of galectin-3 in trophoblast cells were not completely understood. Our qPCR and western blotting results exhibited that high glucose (25 mM) markedly increased galectin-3 expression in trophoblast cells. These results were in agreement with previous studies (Heusler et al. 2021). Heusler et al. (2021) reported that the galectin-3 protein expression level was significantly higher in GDM placental tissue than that in normal pregnancy. Moreover, the results of GSEA showed galectin-3 was closely related to drug metabolism − cytochrome P450, metabolism of xenobiotics by cytochrome P450, complement and coagulation cascades signaling pathways. Cytochrome P450 had a close relationship with glucose metabolism (Xu et al. 2016) and apoptosis (Hua et al. 2013). To explore the effect of galectin-3 on cell apoptosis, HTR-8/SVneo cells were transfected with galectin-3-siRNA under normal glucose (5mM) or moderate glucose (11 mM) or high glucose (25 mM) conditions. Western blotting showed the active caspase-3 and active caspase-3/caspase-3 ratio were significantly increased under high glucose (25 mM) condition, after knockdown of galectin-3. Similarly, flow cytometry analysis showed both early and late apoptosis significantly elevated after the treatment of galectin-3 siRNA. Our findings demonstrating that galectin-3 antagonized high glucose − induced trophoblast cells apoptosis were in line with previous studies (Wesley et al. 2021; Harazono et al. 2014; Yoshii et al. 2002). In fact, some evidence (Saksida et al. 2013; Xue et al. 2019) also shown galectin-3 could perform a pro-apoptotic role under certain circumstances. Therefore, more researches were expected to illustrate the roles of galectin-3.

Foxc1 was a transcription factor that played important roles in apoptosis (Li et al. 2019), cell proliferation (Li et al. 2019), cellular migration (Xia et al. 2013), and cancer progression (Lin et al. 2017). To date, the biological functions of foxc1 needed further research. The results of PPI networks showed the interactions between galectin-3 and foxc1. Furthermore, GSEA indicated that foxc1 was correlated with type I diabetes mellitus pathway. The above results implied that there might be a possible regulatory relationship between galectin-3 and foxc1. Furthermore, to verify the regulatory relationship between galectin-3 and foxc1, in vitro experiments were further performed. Western blotting and qPCR analysis showed high glucose (25 mM) conditions and galectin-3 both upregulated foxc1 expression. Western blotting showed the active caspase-3 and active caspase-3/caspase-3 ratio were significantly increased under high glucose (25 mM) condition, after knockdown of foxc1. Further analysis by flow cytometry revealed early apoptosis and total apoptosis (early plus late apoptosis) were significantly induced in foxc1-siRNA treatment group, compared with that in negative control group. Our results were in line with previous publication (Li et al. 2019). Li et al. (2019) showed that silencing of foxc1 significantly induced apoptosis, while overexpression of foxc1 significantly reduced apoptosis. Recent studies (Zhang and Zhao 2021; Ji et al. 2020) had shown that high glucose (25 mM) significantly promoted apoptosis in HTR-8/SVneo cells. Furthermore, it was noteworthy that a recent study (Cao and Zhang 2022) showed that high glucose reduced the expression of foxc1 in HTR-8/SVneo cells. This study differed somewhat from ours, but there were some important distinctions between the two studies that might explain this discrepancy. Cao S et al. (Cao and Zhang 2022) mentioned “high-glucose (HG) treatment in this study referred to adding extra 25 mM glucose (Sigma-Aldrich) into the normal culture medium (5 mM glucose)”. However, in our study, cells were cultured in normal medium, and then medium was changed to new medium containing 5 mmol/L glucose for 24 h before experiment began. After that, cells were cultured in medium with different glucose concentrations (such as 5 mmol/L, 11 mmol/L, and 25 mmol/L) for 48 h. Also, there were several important differences in included populations between our study and the study of Cao S et al. (Cao and Zhang 2022), such as the OGTT values (OGTT_0, OGTT_60 and OGTT_120). Taken together, our findings were consistent with and extended the results of previous studies and had suggested galectin-3/foxc1 pathway protected HTR-8/SVneo cells against high glucose − induced apoptosis.

Notably, we found that galectin-3 protein expression was negative in STB, where galectin-3 protein expression was observed abundant in CTB, which was consistent with previous literature (Freitag et al. 2020a). The results of single-cell RNA-seq analysis of human placenta in our research group also showed that the expression of foxc1 in the HIP group was significantly higher than that in the HP group in CTB cells (data not shown). CTB cell was an essential constituent cell of the placenta, its dysfunction posed great threats to maternal and fetal health (Zhou et al. 2013). Together, our results indicated that galectin-3 and foxc1 both together were overexpressed in CTB cells, suggesting galectin-3 and foxc1 might play important roles in CTB cells and deserved further study.

Inevitably, there were some limitations in our research and needed to be considered in the future research. First, to the best of our knowledge, this was the first study to analyze plasma galectin-3 levels in GDM group from early pregnancy to delivery. However, the research population of this study was only Chinese pregnant women, these results needed to be investigated in different ethnic populations. Second, even though some studies had suggested galectin-3 promised to be a potential predictive biomarker with satisfactory accuracy, further studies were necessary. We are trying to collect samples in a more large-scale cohort to strengthen our ELISA results. Third, we only used vitro experiments to investigate molecular mechanisms, the data from galectin-3 knockout mice would provide more conclusive results. Fourth, the roles of galectin-3 and foxc1 in CTB cells required further investigation.

## Conclusions

In conclusion, we found significantly higher plasma galectin-3 levels in patients with GDM than that in healthy pregnant women during the first and second trimesters, which filled the gap in the literatures. Our findings suggested galectin-3/foxc1 pathway protected HTR-8/SVneo cells against high glucose − induced apoptosis, and provided a basis for understanding the effects of hyperglycemia on placenta.

### Electronic supplementary material

Below is the link to the electronic supplementary material.

Supplementary Fig. 1. Plasma galectin-3 levels of cord blood in gestational diabetes mellitus (GDM) and healthy pregnant women (HP) groups.

Supplementary Fig. 2. Knockdown efficiencies of foxc1 siRNAs.

Supplementary Fig. 3. The percentages of apoptosis (early apoptosis, late apoptosis, and early apoptosis plus late apoptosis) of HTR-8/SVneo cells were measured by flow cytometry after treatment with foxc1-siRNA-516.

Supplementary Table 1. Primer sequences used in RT-qPCR.

## Data Availability

Publicly available datasets were analyzed in this study. This data could be found here: https://www.ncbi.nlm.nih.gov/geo/query/acc.cgi?acc=GSE154414.

## Abbreviations

BMI: Body mass index
CTB: Cytotrophoblast
GDM: Gestational diabetes mellitus
GSEA: Gene set enrichment analysis
HIP: Hyperglycemia in pregnancy
HP: Healthy pregnant women
IHC: Immunohistochemistry
KO: knock-out
OGTT: Oral glucose tolerance test
PPI: Protein–protein interaction
qPCR: Quantitative polymerase chain reaction
siRNA: Small interfering RNA
STB: Syncytiotrophoblast
