# Supplementary material for: Elevated galectin-3 levels detected in women with hyperglycemia during early and mid-pregnancy antagonizes high glucose − induced trophoblast cells apoptosis via galectin-3/foxc1 pathway
Source: Mol Med. 2023 Aug 25;29:115. doi: 10.1186/s10020-023-00707-5 (PMC10463409; doi:10.1186/s10020-023-00707-5)
Supplement: Supplementary file 1 — Supplementary Fig. 1. Plasma galectin-3 levels of cord blood in gestational diabetes mellitus (GDM) and healthy pregnant women (HP) groups. [file 10020_2023_707_MOESM1_ESM.docx]

**Supplementary materials**


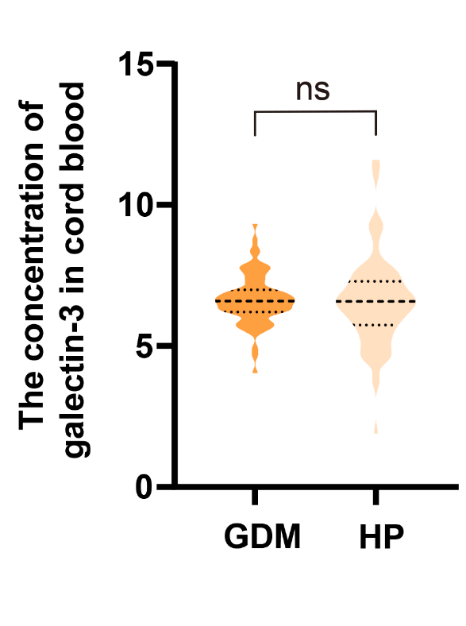


**Supplementary Figure 1.** Plasma galectin-3 levels of cord blood in gestational diabetes mellitus (GDM) and healthy pregnant women (HP) groups.
