# Supplementary figures and images for: Elevated galectin-3 levels detected in women with hyperglycemia during early and mid-pregnancy antagonizes high glucose − induced trophoblast cells apoptosis via galectin-3/foxc1 pathway
Source: Mol Med. 2023 Aug 25;29:115. doi: 10.1186/s10020-023-00707-5 (PMC10463409; doi:10.1186/s10020-023-00707-5)

**Supplementary materials**


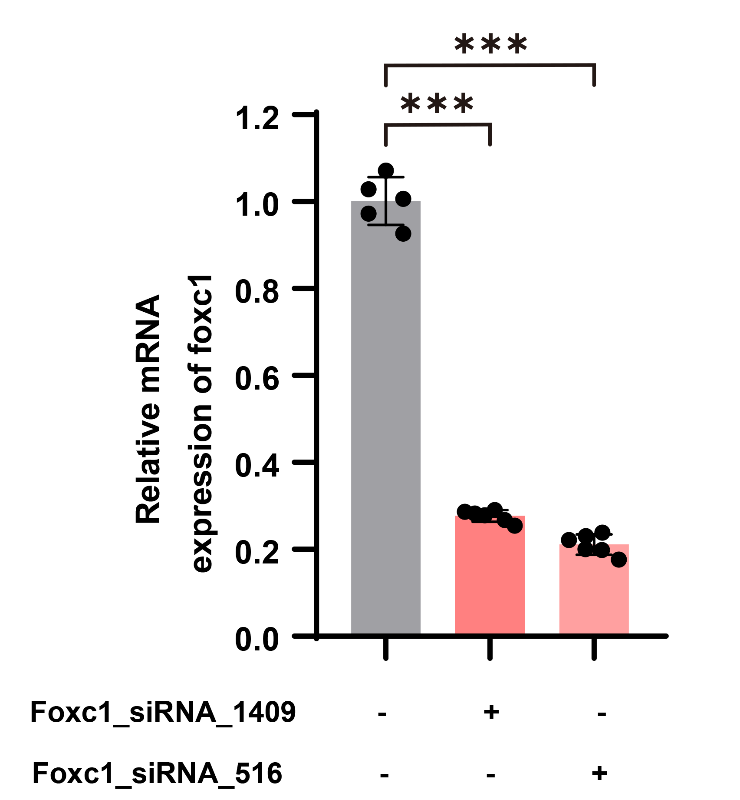


**Supplementary Figure 2.** Knockdown efficiencies of foxc1 siRNAs.

Supplement: Supplementary file 2 — Supplementary Fig. 2. Knockdown efficiencies of foxc1 siRNAs. [file 10020_2023_707_MOESM2_ESM.docx]
