# Supplementary material for: Elevated galectin-3 levels detected in women with hyperglycemia during early and mid-pregnancy antagonizes high glucose − induced trophoblast cells apoptosis via galectin-3/foxc1 pathway
Source: Mol Med. 2023 Aug 25;29:115. doi: 10.1186/s10020-023-00707-5 (PMC10463409; doi:10.1186/s10020-023-00707-5)
Supplement: Supplementary file 3 — Supplementary Fig. 3. The percentages of apoptosis (early apoptosis, late apoptosis, and early apoptosis plus late apoptosis) of HTR-8/SVneo cells were measured by flow cytometry after treatment with foxc1-siRNA-516. [file 10020_2023_707_MOESM3_ESM.docx]

**Supplementary materials**


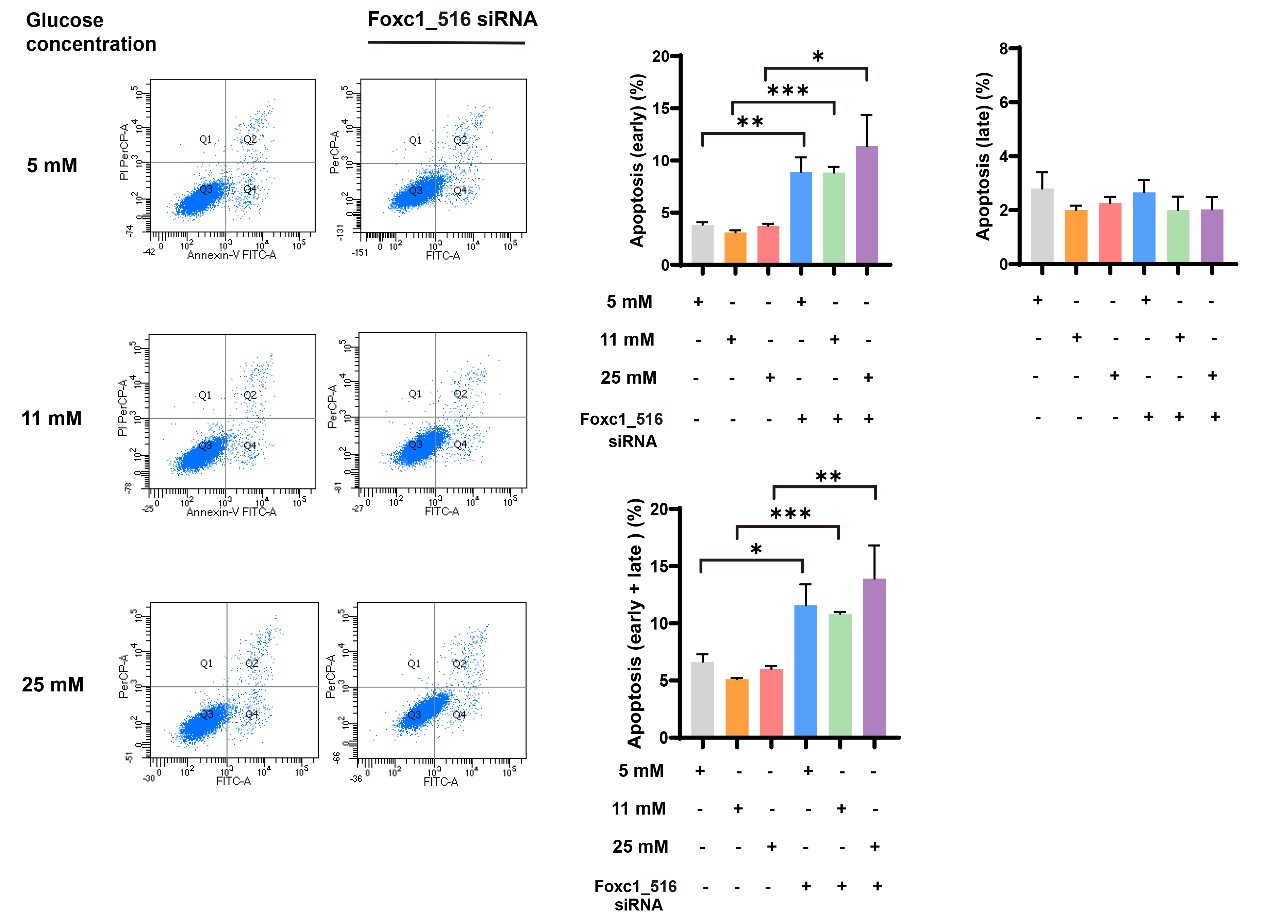
 **Supplementary Figure 3.** The percentages of apoptosis (early apoptosis, late apoptosis, and early apoptosis plus late apoptosis) of HTR-8/SVneo cells were measured by flow cytometry after treatment with foxc1-siRNA-516. Q1, necrotic cells; Q2, late apoptosis cells; Q3, living cells; Q4, early apoptosis cells. *: *P* < 0.05, **: *P* < 0.01, and ***: *P* < 0.001.
