# Supplementary material for: Elevated galectin-3 levels detected in women with hyperglycemia during early and mid-pregnancy antagonizes high glucose − induced trophoblast cells apoptosis via galectin-3/foxc1 pathway
Source: Mol Med. 2023 Aug 25;29:115. doi: 10.1186/s10020-023-00707-5 (PMC10463409; doi:10.1186/s10020-023-00707-5)
Supplement: Supplementary file 4 — Supplementary Table 1. Primer sequences used in RT-qPCR. [file 10020_2023_707_MOESM4_ESM.docx]

**Supplementary materials**

**Supplementary Table 1.** Primer sequences used in RT-qPCR

| **Genes** | **Forward primer/Reverse primer** | **Cite** |
| --- | --- | --- |
| Galectin-3 | 5’-CCATTTGAAAGTGGGAAACCA-3’  5’-CATCATTCACTGCAACCTTGAAG-3’ | PMID: 30246456 |
| Foxc1 | 5’-CGGCTTGAACAACTCTCCAG-3’  5’-ACAGTCGTAGACGAAAGCTCC-3’ | PMID: 28287613 |
| GAPDH | 5’-TGCACCACCAACTGCTTA-3’  5’-GGATGCAGGGATGATGTTC-3’ | PMID: 34245854 |
